# LBTM: Listen-before-Talk Protocol for Multiclass UHF RFID Networks

**DOI:** 10.3390/s20082313

**Published:** 2020-04-18

**Authors:** Pablo López-Matencio, Javier Vales-Alonso, Juan J. Alcaraz

**Affiliations:** ICT Department, Technical University of Cartagena, 30202 Cartagena, Spain; javier.vales@upct.es (J.V.-A.); juan.alcaraz@upct.es (J.J.A.)

**Keywords:** RFID networks, LBT, traffic, time slot

## Abstract

Radio Frequency Identification (RFID) is considered one of the pioneering technologies of the Internet of Things (IoT). It allows to bind physical environments to information processing systems, adding new capabilities like automatic inventorying, location, or sensing with batteryless tags. Indeed, many data flows of physical objects can be tracked using this technology, and it is common to find heterogeneous traffics present in the same facility, each managed by different sets of readers. For example, in a grocery store, typically we have two kinds of readers: those carrying out a continuous inventory, whose goal is knowing the contents of the shelves as accurately as possible; and a set of checking-out readers at exit gates for the billing process that has to minimize the waiting time of customers. Another example of multiclass traffic is a hospital, where new families of sensing tags allow staff to wirelessly monitor patients—which obviously must be done as a priority—and coexist with other readers aimed at precisely knowing the location of equipment or drugs. Even with the same goal, there could be readers requiring different setups, for example in the hospital case, readers located at doors for inventorying purposes have a short time available to identify passing-by objects or people, and thus they have to work with a higher priority than regular readers performing inventorying tasks. In this work, we investigate a modification of the standard listen-before-talk (LBT) protocol for RFID networks which can support this kind of multipriority environment, by offering different qualities of service to each traffic. Results demonstrate that by tuning the protocol setup, it is possible to establish a trade-off between the performance of each traffic. This is shown for the two cited examples, the grocery shop and the hospital, using a simulation tool allowing us to implement a full-scale RFID model. In addition, we present a greedy mechanism for online reader setup. Instead of selecting offline a hard priority level, this greedy algorithm is able to adapt the priority to achieve the required quality-of-service (QoS) level.

## 1. Introduction

Multireader RFID deployments (also known as RFID networks) can be subject to constraints imposed by the different nature of the traffics accessing each reader. For example, in the same facility, some readers may work in the background doing a low-priority, continuous inventory process, while others may be serving the customers’ checking-out processes and have a higher priority. These systems are said to have multiclass traffics, and specific solutions may be used to balance the performance of each class. For example, in the previous case, the customers’ class traffic waiting times can be reduced at the expense of a more imprecise continuous inventory process. These types of trade-offs will be required in actual environments, where readers access heterogeneous sources of tags.

RFID networks experience reader-to-reader interferences since neighbor reader signals easily jam the weak tags’ responses. Therefore, it is necessary to arbitrate access to the shared interrogation channel using different mechanisms. The standard EPCglobal Class 1 Gen2 [1] introduces Multiple-Interrogation-Mode (MIM) or Dense-Interrogation-Mode (DIM), which alleviate the reader-to-reader interferences by making the tags respond in the sidebands of the readers’ transmission channels.

Another approach is using listen-before-talk (LBT), which senses the channel for an interval to determine if it is free before proceeding with the transmission. Otherwise, it defers its transmission to a later time. Depending on the country’s regulations, LBT can be regarded as an optional mechanism for RFID networks. The EPC Global report [2] shows that it is in use in Japan, South Korea, Latvia, and the Russian Federation, while other countries use frequency-hopping (FH) [3] or the ETSI normative (ETSI 300-220 [4]), whose polite-sprectrum-access (PSA) mode is a type of LBT.

In this paper, which is an updated and enhanced version of our previous work [5], we propose a simple variation of the LBT protocol, named LBTM (LBT Multiclass), to handle multiclass traffics in RFID environments. When a reader tries to occupy the channel, it has to wait a fixed listen time (LT)—plus a random contention time (CT) if the channel is busy—until the channel is free. Then, the reader can start the interrogation of the tags nearby. The core idea of LBTM is to modify the access loop if the channel is busy by waiting Δ LT periods until probing the channel again, Δ being a parameter that depends on the traffic class and that is higher for the lower priority classes. In short, a higher Δ allows expanding the back-off period for low-priority classes, and a relatively lower Δ to prioritize traffic.

This operation still allows for transmission opportunities for the lowest class readers, since after the back-off period, all readers behave similarly. Therefore, LBTM only provides soft QoS guarantees (i.e., statistical bounds), rather than hard QoS limits achieved with other disciplines like the preemptive head-of-the-line (HOL) one [6]. On the other hand, LBTM is limiting the starvation of the lowest class traffics, thus being suitable for heterogeneous environments.

In this work, we aim at describing the operation of LBTM and studying how to set up and characterize a realistic network layout. Conceptually, the operation of the LBTM mechanism in a network can be considered as a single-server system (if all readers are mutually interfering, the channel is a resource that can only be accessed by one reader at a time) with separate traffic queues per traffic class (*polling model*) and finite-users, since the number of readers of each class is finite. The queue discipline is Random Order of Service (ROS) since users are served at no particular order but randomly (see, e.g., Chapter 2 in [7] and references therein). Besides, interarrival and service times are non-exponential. These types of polling models are complex to analyze and would require multiple simplifications, yielding a lack of results’ reliability. Therefore, we are studying LBTM performance through a simulated environment developed in Netlogo [8]. All experiments and code developed in this work are publicly available for reproducibility purposes at the Github authors’ public repository (https://github.com/plopezmp/LBTM).

Our experiments show that, with a proper selection of the number of listening periods (the Δ’s), it is possible to accommodate each traffic to its specific requirements. For example, for the grocery shop example, it is easy to achieve a faster checkout process for customers keeping a similar accuracy ratio in the inventory process. For the case of the hospital, it is possible to achieve a balance point where the number of interrogation cycles per second is suitable for the selected initial goals.

Additionally, we also address the problem of deciding online which is the best Δ (priority class) that readers have to use to achieve a required QoS, which is modeled by using a utility function. The algorithm runs independently in each reader and has a greedy operation, increasing the Δ whenever the utility is below a given threshold. Results show that this online mechanism achieves close to optimal performance for the example of the hospital (comparing it to the optimal one obtained offline).

The rest of this work is organized as follows: Section 2 reviews the related literature on multiclass traffic control in the context of RFID networks. Section 3 describes the LBTM protocol, while Section 4 describes the simulator framework used. The grocery shop example is then analyzed in Section 5, while the hospital example is described in Section 6. Section 7 discusses the greedy mechanism for online network setup. Section 8 provides a comparison between LBTM+DIM and DIM standalone mode. Finally, Section 9 summarizes our main findings and future work.

## 2. Related Works

Contention protocols arise in many practical communication networks, and in most cases, they consider homogeneous nodes and traffics between them. For example, the well-known nonpersistent carrier-sense multiple access (CSMA/CD) that uses a back-off algorithm, such as that employed in local area networks [9], is an example of this kind. However, legacy CSMA/CD does not support service differentiation or priority access to the channel. The work [10] presented an analytical approach to this problem by assigning different durations for the contention windows to each traffic class. This idea is similar to LBTM, although in LBTM, only the first contention window adds additional back-off time to avoid starvation of the lowest priority readers.

Other important back-off approaches incorporating multiclass differentiation are *Predictive* [11], *Sliding Contention Window* [12], *Gentle DCF/Probabilistic* [13], *Polynomial Backoff* [14], and *Fibonacci Backoff* [15] schemes. With the exception of [10,14], these proposals show significant degradation when the number of contenders rise up. In the case of *Fibonacci Back-off* [15], the authors focused only on tag collisions and did not address reader-to-reader collisions.

None of the previous approaches have been used or proposed yet for an RFID network. Most of the earlier algorithms can be adapted to some extent to work in this context. However, it would be necessary to do performance studies since the traffic characteristics of an RFID environment are quite different from those considered in the related works. This is indeed the primary goal of our work.

On the other hand, several works have dealt with different traffic-load-balancing issues in RFID networks, but are mainly focused on readers positioning or other aspects unrelated to the reader-anti-collision protocol. Works of this type are [16,17,18,19,20,21]. None of these proposals provide mechanisms to share the channel during operation. One recent mechanism with this aim was introduced in [22], which presented a distributed reader-to-reader anti-collision protocol based on Petri nets to assign interrogation time slots to the different readers—although, as with most works on RFID, the authors did not consider multiclass traffics.

Random-access protocols aimed at multiclass RFID or general-purpose Internet of Things (IoT) networks are scarce. To some extent, works exist for machine-to-machine (M2M) cellular networks, like in [23], where authors introduce a Time Division Multiple Access (TDMA) protocol for heterogeneous traffic in 3GPP Long Term Evolution networks. More similar to our environment, in [24], authors study the application of Aloha protocol for multiclass networks. They approximate the solution by maximizing a global utility function designed as a weighted sum of all classes’ utility functions. The solution found assures maximum utility when the higher priority classes have a higher probability of accessing the medium. In our approach, we also design a global utility function to permit the selection of the optimal network operation point. In [25], authors propose an irregular repetition slotted Aloha (IRSA) [26] scheme consisting of transmitting packet replicas and exploiting interference cancellation at the receiver for achieving higher throughput. However, this kind of multitransmission scheme is not suitable for RFID networks, where communication between readers and tags has to be free of interfering sources.

## 3. LBTM Operation

Following the 2016 version of the the ETSI EN 302-208-1 standard [27], we assume a maximum interrogation time (IT) of 4 s, and minimal waiting time between two consecutive ITs of at least 100 ms. The access to the channel [28] operates as follows:

1. Before occupying the channel, a reader probes the channel during a listen period of 5 ms. Henceforth, this period is termed as *listening time* (LT).
2. If the channel is free during this time, the reader can start the interrogation process.
3. If the channel is busy, it waits for a contention period between 0 and 5 ms selected at random (in 11 discrete values).
4. It repeats the random time until the channel is free, and then it starts the interrogation.

The LBTM protocol uses this same structure, and the single difference appears in point 3, where a back-off time of duration $\Delta_{j}$ LT periods (including the first LT) is inserted before the contention periods, where *j* indicates the particular traffic class, being *j* = 1, …, *C*, with *C* being the total number of classes. Note that the back-off period has a fixed length for each class. This way, when several readers of different classes are waiting for the channel, it is more likely that a high-priority one occupies it after it becomes idle. Moreover, after the corresponding back-off period, readers access the channel with the same priority. This way, starvation of the lower priority ones is avoided.

Finally, an additional reason for keeping the LBTM protocol simple is to facilitate its implementation in actual readers already supporting LBT [29]. With LBTM, a reader would require a single additional configuration parameter (the Δ associated with its traffic). Here, we assume that each reader is associated exclusively with a fixed class, although possible variations where the reader can autodetect the underlying traffic class dynamically could also be considered. We describe an online possibility in Section 7. Note that different readers may have the same underlying traffic and can be assigned the same Δ.

**Figure 1:**
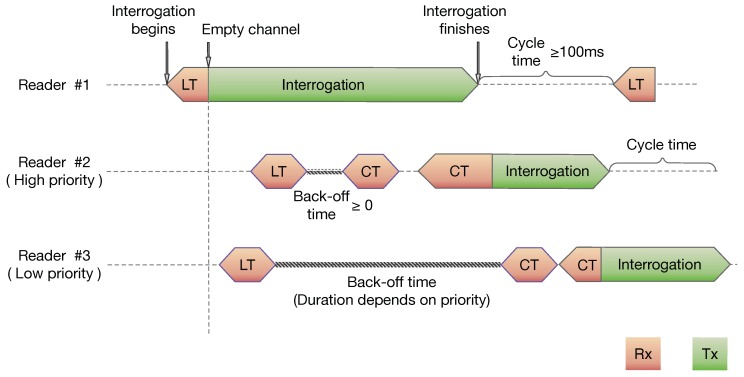
Listen-before-talk multiclass (LBTM) operation. The upper timeline shows Reader 1, which finds the channel empty and starts interrogation immediately regardless of its priority. If the channel is busy, then the readers have to wait a back-off time, which is higher for lower priority classes. Reader 2 waits a short back-off time (potentially 0 for the highest class), while Reader 3 has to delay its channel access for a longer period. After back-off time, all readers contend in similar conditions, avoiding low-priority class readers’ starvation.

An example of the operation of LBTM is shown in Figure 1, for a *C* = 2 classes configuration with 3 readers. When the channel is idle, regardless of its class, the reader just uses the channel (Reader 1 in the first row of figure). If the channel is busy, a short back-off time (low Δ) is used for the high-priority traffic class (middle row in Figure 1), whereas the low-priority traffic shown below has a long back-off time (high Δ). As can be seen, back-off periods delay the access of the low-priority reader which—instead of starting its interrogation process earlier—has to delay it until after the high-priority reader finishes.

## 4. Simulation Framework

LBTM has been analyzed using the simulator framework described at [30], which is publicly available for download at [31]. The particular setup used in this paper is available in the link provided in the introduction.

**Figure 2:**
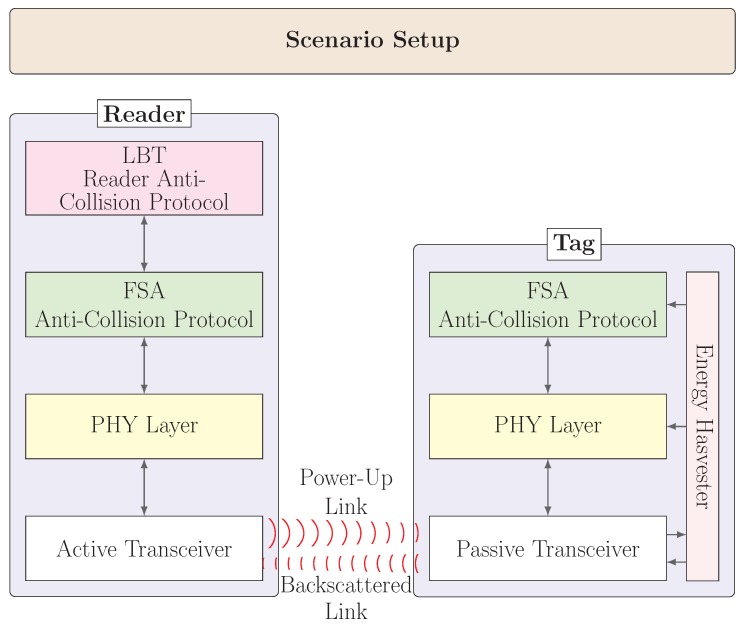
Simulator protocol stack design.

The protocol stack implemented is shown in Figure 2, and the main features of the simulator are the following:

- It uses a detailed and accurate link budget model, considering distance, antenna aiming, multipath propagation, and shadowing effects. The main physical parameters are summarized in Table 1.
- The location of gates, tags, and all the physical elements can be changed, including dynamic motion.
- It implements several tag anticollision protocols. In particular, in this work, we use the well-known Dynamic Frame Slotted Aloha (DFSA) approach with perfect tag-count estimation. This protocol adapts the interrogation frame length to the number of tags contending to achieve an optimal throughput (e.g., [32] and references therein). Static FSA is also feasible by setting a correct frame size suitable for the incoming traffic.
- It allows multiple traffic patterns, including batch-traffic, continuous-flow traffic, and permanent load. Therefore, it is possible to characterize accurately different real-world situations.
- Finally, it is flexible enough to implement any reader anticollision protocol on top of its stack. By default, it uses LBT/LBTM, but it is possible to update it to include other synchronous or asynchronous schemes.
- The interrogation process lasts until no more tags respond to the readers’ Query packets.

**Figure 3:**
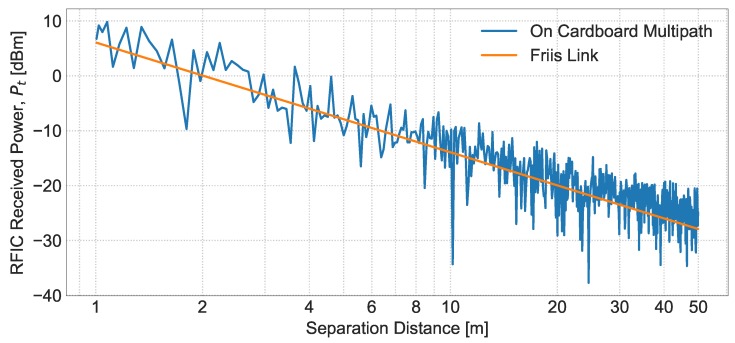
Example of the power received by the tags using two different link budget setups in the simulator, one considering the Friis model and the second assuming a cardboard substrate for the tags.

The simulation framework computes the signal to interference and noise ratio (SINR) individually for each tag transmission. It considers the full link budget between the source or the interfering element and the reception point (as an example, Figure 3 shows the received power for two types of setups available in the simulator). The interference can come from other tags or readers, depending on the type of the tag and reader anticollision protocols and on the particular event. However, note that LBT/LBTM avoids simultaneous transmissions except in the case of readers’ collisions. Besides, it is worth mentioning that, in contrast to most wireless systems, RFID systems commonly have the capacity of detecting collisions since the transmission and reception signals are associated with different antennas, although the standard does not preconfigure this feature as mandatory. Thus, in LBT and LBTM, it is certainly possible to implement the Collision Detection mechanism, and the simulator considers this mechanism by default.

In the next sections, a detailed layout of each simulated scenario is described, as well as the traffic characteristics and the key performance indicators (KPIs).

## 5. Grocery Shop Example

In our first example, we have considered a setup with only two types of traffic, closely resembling a grocery store scenario like the one described in [33]. These are described as follows:

- **Inventory traffic**. A set of readers realizes a continuous inventory of the items distributed in the store area. The goal of these readers is to maximize the ratio of items correctly inventoried. This metric is called MSL (Minimum Stock Level) [34], and it measures inventory accuracy. MSL is defined as the time-average ratio of time where the percentage of known tags divided by the total number of tags is over a given minimum threshold. For example, an MSL with a threshold of 90% and accuracy of 99% indicates that in at least 99% of the queries (done at randomly chosen time moments) to the inventory database, 90% of the reported data will be exact. Note that items constantly appear, due to replacement tasks; or disappear, if they are picked up by customers. Thus, yielding to frequent changes in the inventory.
- **Checking-out traffic**. This represents the customers leaving the store. In this case, the customers wait in a shared exit queue until a checking-out gate is available. The number of tags per customer is random, and the batch for each particular buyer is read independently. This traffic has a high priority, and the goal is to minimize the total response (waiting plus interrogation) time of the clients.

**Figure 4:**
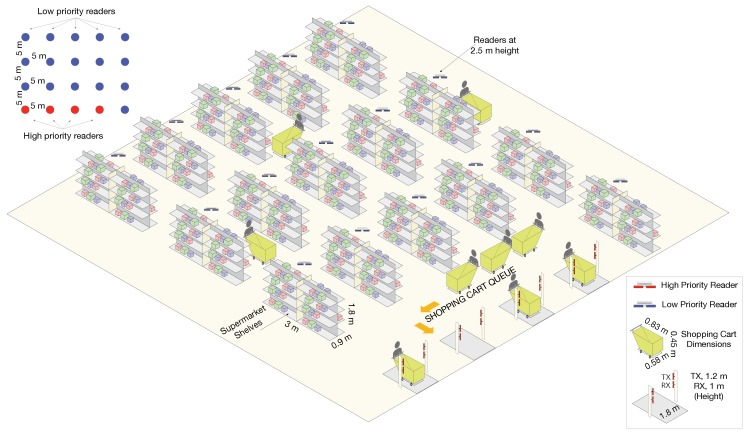
Configuration scheme of the readers and checkouts in the supermarket. Antennas are at 1-m and 1.3-m height at checkouts, and 5-m height above the floor of supermarket shelves. Checkouts are RFID portals of 2-m width to let carts pass through. Each stock inventory reader comprises a reader and 4 shelves of 0.9 m × 3 m × 1.8 m (W × L × H) placed back-to-back pairwise.

The simulation layout is shown in Figure 4. It consists of 4 easy-pass checking-out gates and 16 ceiling readers configured with the following parameters:

- The inventory cycle time for the ceiling readers is 15 s. That is, the time between two consecutive interrogation processes of a ceiling reader.
- The checking-out readers have the minimal cycle time allowed by the ETSI EN 302-208-1 standard [27] of 100 ms.
- Each shelf in the store may contain up to 250 items (tags), and each ceiling reader manages four shelves. Therefore, a total number of up to 1000 tags have to be read at each interrogation process.
- The number of items (tags) picked by each customer is a random variable with a mixed Poisson-lognormal (PLN) distribution [35], which has been found to model this kind of process correctly.
- The clients arrive at the exit queue at a rate of λ=1 customer (batch) per second.
- Simulation stops after 4000 clients have left the store.
- The replacement task is performed every 15 min in a random number of shelves (between 1 and 4).
- When a customer leaves the store, the same number of items (tags) as those in its batch are selected at random and removed from the shelves.
- All readers cause mutual interferences. In the best case, the minimum interference corresponds to two readers installed in the ceiling at 2.5 m height and which are separated by almost 29 m. The interference signal is reflecting in the ground and is received with a power of slightly below −23 dBm, much higher than the tags’ backscattered signals.

### Results

**Figure 5:**
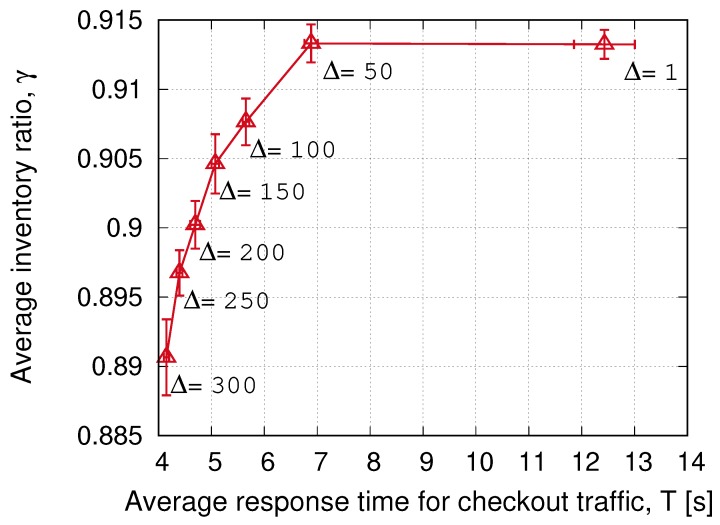
Pareto-front for the trade-off between checking-out and inventory traffic performances for the grocery shop scenario, as a function of Δ. Each point is the average of 20 simulations. The confidence interval is shown with 95% confidence level. The MSL threshold is 80%.

Figure 5 shows the accuracy of the inventory process with an MSL threshold of 80% versus the response time (waiting time in queue plus interrogation time) for the checking-out traffic. At each point, the Δ parameter is changed with the values indicated in the figure. Three primary outcomes are clear from these results:

- A very significant reduction of the response time can be achieved with a negligible loss in the inventory accuracy by selecting Δ = 50.
- Higher Δ values can trade-off even less response time for minor losses in the accuracy. The reason for this minimal accuracy loss is that the inventory is a slow-varying process. A significant number of items have to be incorporated or removed to have a noticeable accuracy change, and the readers will be most likely able to correct the dataset, even with low-priority access to the channel.
- Pareto-front shows an exponential shape, and we can effectively select a response-time goal (only limited by the interrogation time, which does not depend on the Δ parameter).

**Figure 6:**
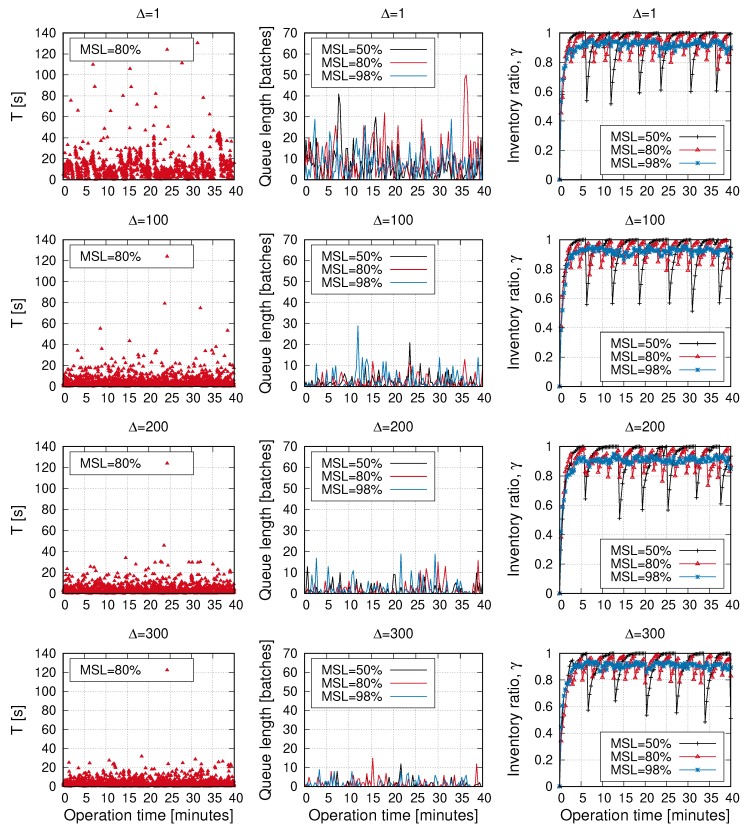
Time-evolution of the response time (**left column**), the number of customers in the exit queue (**middle column**), and the inventory ratio (**right column**) for a simulator realization versus Δ.

On the other hand, Figure 6 provides additional insight in LBTM operation. It shows the time evolution of the response time (left column), the number of customers in the exit queue (middle column), and the inventory ratio (right column) for a simulation. Each row represents the results for a given Δ parameter. The first (upper-most) row corresponds to the standard LBT behavior. The queue backlogs and the response times have a higher variance for the lower Δ values, as the checking-out readers have to struggle with the inventory process ones more often. As the Δ values increase, the contention for the channel is usually constrained to checking-out readers, which can serve the traffic quickly and avoid customers having to wait in the exit queue, as can be seen from the reduced backlogs.

## 6. Hospital Example

### 6.1. Background

For the second example, we focus on the use of RFID in a hospital environment. We have selected this example due to the significant implantation of RFID technologies in the health sector. Different reports have discussed the potential uses in this sector. For example, Fuhrer et al. [36] cited the following, among other applications:

- Patient identification;
- Blood samples tracking;
- Security on sensitive surgical equipment and pharmacy;
- Tagging meals to ensure patients get the appropriate diet according to their treatment, allergies, and tastes;
- Laundry service can also be greatly optimized;
- Entrance control.

More recently, Haddara et al. [37] analyzed the different applications and provided useful references for discussing each of them. The main areas they cover are as follows:

- Pharmaceutical/blood product distribution and tracking;
- Patient/medical staff identification and tracking;
- Medical asset tracking and locating;
- Implantable device RFID use;
- Other areas (including medical documents and patient records) (Kalorama, 2010).

A cost-benefit analysis of some of these applications has been discussed in [38]. Besides, several works have studied how to implement novel applications for the healthcare sector based on RFID. In [39], the authors developed a system for apnea detection based on an unsupervised learning scheme. Hou et al. [40] developed a nonintrusive breath monitoring. In [41], researchers described the design and implementation of cabinets with RFID support to improve the tracking of medical supplies in hospitals. The authors of [42] proposed using RFID for two tasks: (i) management of bottle gas delivery, whose cost can be endorsed to a particular area or another hospital if the patient is transferred; and (ii) tracking ancillaries and surgical instruments, for example, to know when they have been sterilized. Patients tracking has been analyzed in different works such as [43] or [44]. Moreover, different companies like IGSolutions [45] are offering RFID solutions for the inventory and tracking of controlled substances.

Although promising, certain RFID applications have faced resistance in a hospital setting where their electromagnetic interference could affect normal operation of medical instruments (e.g., [46,47]). However, despite these issues, RFID technology has great potential in healthcare applications. In this context, it is mandatory to reduce the number of channels and power used by RFID readers to ensure electromagnetic compatibility [38,48,49], while the available channel has to be used efficiently among several RFID services.

### 6.2. Scenario Layout

**Figure 7:**
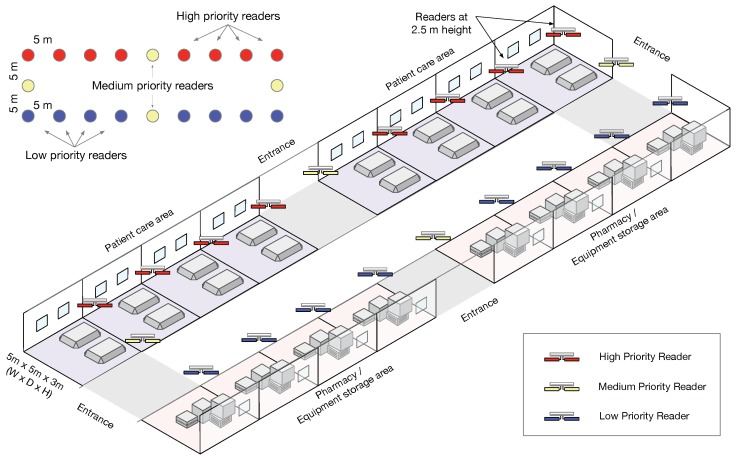
Hospital layout.

The layout considered for the hospital models a single floor with several rooms for patient care as well as several rooms for storage of equipment and drugs, as shown in Figure 7. We assume that patients can be tagged for knowing their location or for other uses like breath monitoring, such as in [40]. Besides, items or drugs are also tagged for continuous inventory. Finally, this layout has several entrances to control people and items passing by. The key performance indices selected in this example are the number of inventories (cycles) per second that each reader type can do. The readers for patient monitoring will have the highest priority, followed by the readers at doors due to the limited identification time for this traffic, while those readers doing a continuous inventory of items inside rooms have the lowest priority. In addition, the next setup has been selected:

- There are 20 readers installed at 2.5-m height distributed as follows: 8 perform patient monitoring (class 1, highest priority), 8 do continuous inventory of storage rooms (class 3, lowest priority), and the other 4 control items and patients crossing doors (class 2, medium priority).
- Each reader has an initial load of 20 tags.
- Arrival rate at each door is a Poisson of rate 1 group of people every 120 s, with the people in each group also given by a Poisson distribution of rate 3.
- Cycle time is set to the minimum of 100 ms for all reader classes.
- Tagged equipment has a reorganization rate (they change their rooms) every 4 min, involving a random number between 20 to 60 items.
- Simulation stop condition is 1 h of operation.
- As in the grocery shop example, all readers cause mutual interferences; in this case, the minimum level of interference is −26.3 dBm for readers at 42 m.

### 6.3. Results

**Figure 8:**
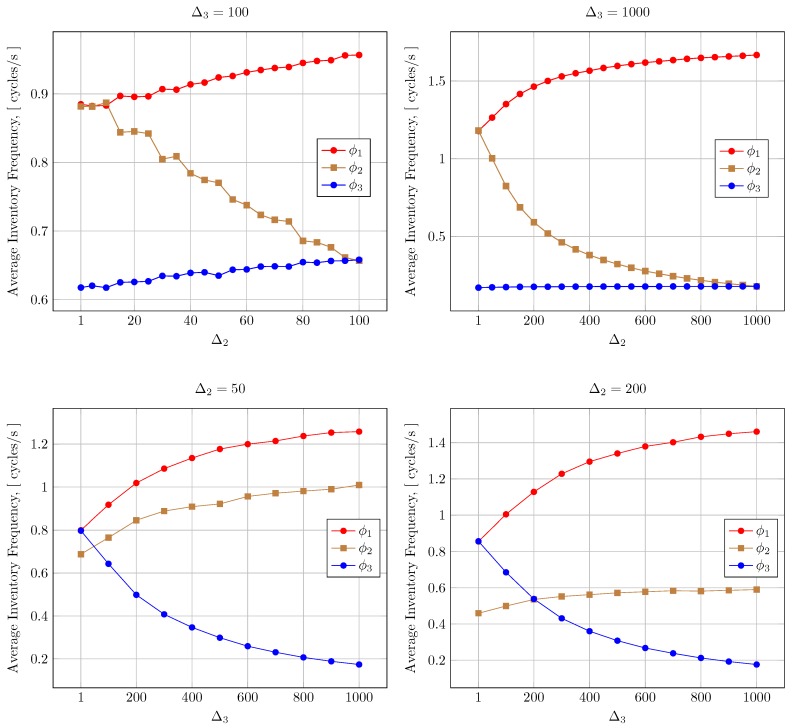
Inventory frequencies for different Δ setups.

Figure 8 shows the number of inventory cycles allocated for each type of reader ($\phi_{1},\phi_{2},\phi_{3}$) for a fixed $\Delta_{1}$ = 1 (that is, no back-off time after the first LT for the patient monitoring readers) and either fixing $\Delta_{3}$ = 100 or 1000 (for the inventorying readers inside the storage rooms) or fixing $\Delta_{2}$ = 50 or 200. The *x*-axis indicates the free Δ parameter.

As with the grocery shop example, the variation of the Δ parameters produces a noticeable change in the system performance, showing a clear trade-off between different traffic types. For example, when $\Delta_{2}$ grows, readers of the first and third class increment their cycles/s (a significant amount in the case of first-class readers). The same occurs when $\Delta_{2}$ is fixed but $\Delta_{3}$ grows, reducing the priority of those readers and increasing the inventory frequency for the rest.

Finally, we have also developed an expression to measure the *utility* of a given configuration, in order to ease the interpretation of the results. This utility function is given by

(1) $$u\left(\phi_{1},\phi_{2},\phi_{3}\right)=\mathrm{sigm}\left(a_{1}\phi_{1}-b_{1}\right)\mathrm{sigm}\left(a_{2}\phi_{2}-b_{2}\right)\mathrm{sigm}\left(a_{3}\phi_{1}-b_{3}\right),$$

where sigm denotes the *sigmoid* function and $a_{j},b_{j}$ for j=1,2,3 are class parameters selected such that the sigmoid has values 0.1 and 0.9 respectively for a given minimum and a maximum number of cycles per second. The idea behind this expression is that for a configuration of Δ’s being “good”, it is required that it achieves a given minimum frequency of operation for each traffic. On the other hand, if the frequencies are too high, no additional benefit is obtained. The sigmoid product allows for capturing this behavior.

The frequencies used for each traffic (see Table 2) as references are the following:

- For patient monitoring readers, the minimum and maximum frequencies are respectively 1 and 1.5 cycles/s;
- For door readers, the minimum and maximum frequencies are respectively 0.2 and 0.5 cycles/s;
- For room-inventorying readers, the minimum and maximum frequencies are respectively 0.1 and 0.2 cycles/s.

**Figure 9:**
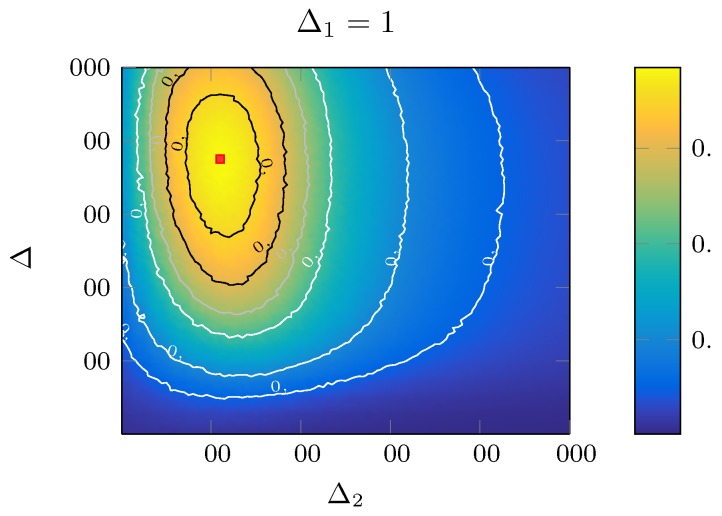
Configuration utility as a function of Δ setups.

For this setup, Figure 9 shows a color map with the resulting utility. Note that once again, $\Delta_{1}$ has been set fixed to 1, and value modifications are allowed for $\Delta_{2}$ and $\Delta_{3}$. Results show that the optimal utility is above 0.76 and this is achieved for $\Delta_{2}$ = 220 and $\Delta_{3}$ = 750.

In summary, this utility function is a powerful *offline* tool for a network designer to select a proper balance for the network configuration. An *online* alternative, allowing the readers to autoselect their Δ parameter, is described in the next section.

## 7. Greedy Online Scheme

Offline setup lacks flexibility adapting to changing traffic conditions or to different network conditions (e.g., new readers). An alternative to this design is allowing each reader to decide *autonomously* and *online* which is the traffic priority that it must establish. The network designer decides the utility limits ($u_{\max},u_{\min}$) for each reader (usually according to the traffic class, as in the previous sections). Then, during operation, each reader checks if the achieved utility is within the target range and corrects its operation.

The algorithm works as follows:

- Each reader operates independently, and for every decision period it estimates its interrogation cycle ϕ = Cycles in the last decision period/Decision period duration.
- The utility *u* is computed from ϕ as sigm(aϕ−b), *a* and *b* being the parameters associated to the particular reader traffic (see Table 2).
- If $u>u_{\max}$, then Δ←Δ+5.
- If $u<u_{\min}$, then Δ←Δ−5.
- Otherwise, Δ is kept fixed.

This algorithm is dubbed as *greedy* since it increments its class unconditionally when the performance is degraded.

**Figure 10:**
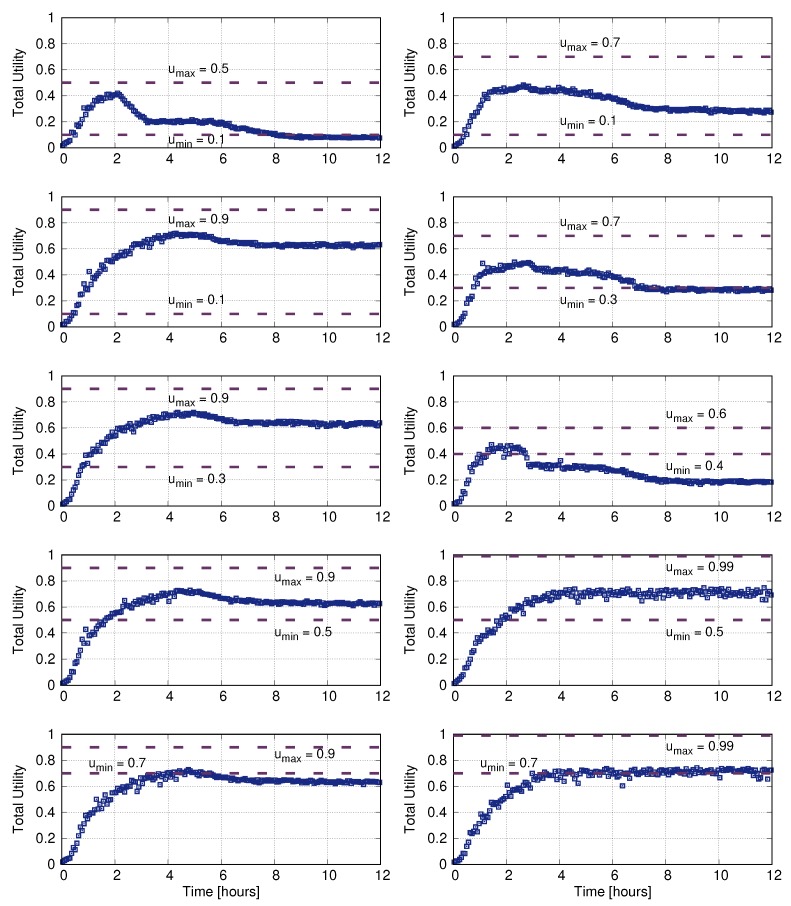
Total utility with the varying min and max decision thresholds.

We have tested this scheme in the hospital environment studied in the previous section, for a decision-period-duration of 120 s, setting different utility limits (but homogeneous for all readers). Figure 10 shows the evolution of the total utility given by Equation (1) for a simulation trace. As shown, the utility limits determine the long-term performance and the stabilization period. The best performance is achieved for a high (close to 1) $u_{\max}$. For lower values, the long-term utility tends to drop from a maximum and then stabilize there. In these cases, the transient period is about 4 h (about 120 decision periods).

**Figure 11:**
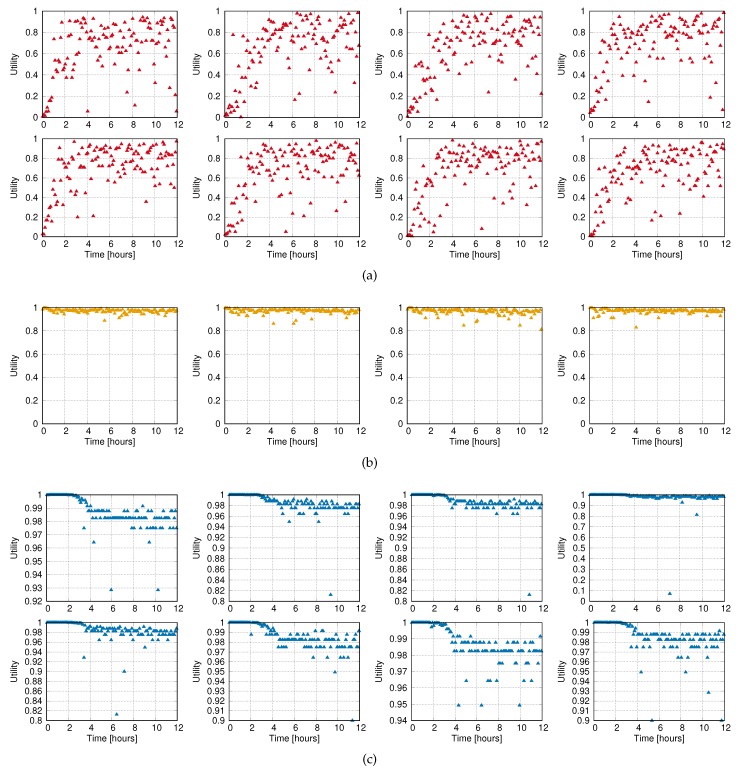
Utility traces. (**a**) High priority readers for patient monitoring. (**b**) Medium priority readers of entrances. (**c**) Low priority readers for storage monitoring.

**Figure 12:**
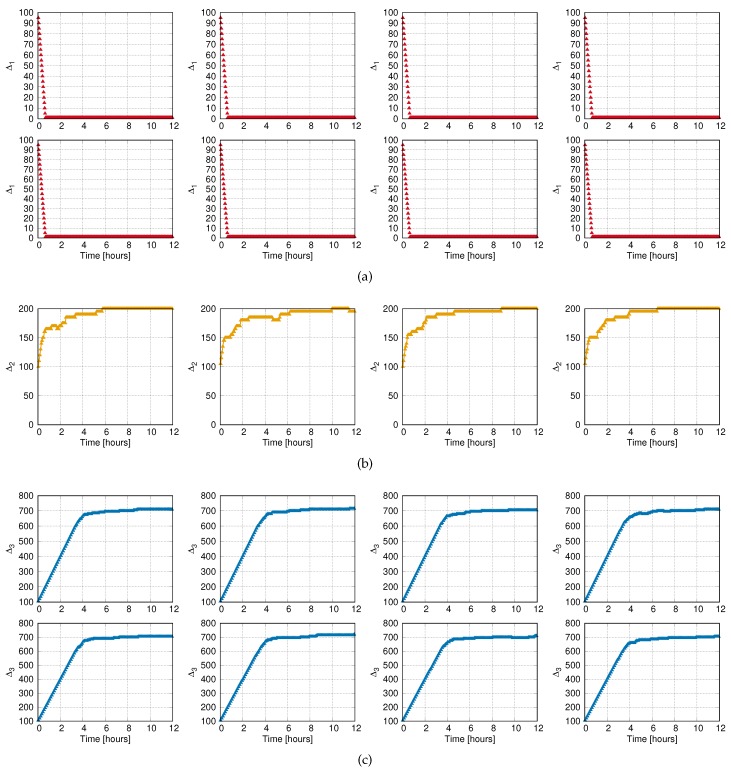
Δ evolution. (**a**) High priority readers for patient monitoring. (**b**) Medium priority readers of entrances. (**c**) Low priority readers for storage monitoring.

Further insight in the online scheme can be seen in Figure 11 and Figure 12. They show respectively the utility and Δ traces for each reader in the hospital setup. As shown, the patient monitoring readers gain utility at the expense of the other groups. The convergence for these high-priority readers is quick (∼15 decision periods), due to the greedy nature of the algorithm. Besides, the medium-priority readers (door readers) steadily increase their Δ but at a slower pace than the low-priority ones (storage inventorying).

## 8. LBTM+DIM Mode Analysis

As described in the introduction, the EPCglobal Class 1 Gen 2 standard [1] uses DIM and MIM modes to deal with reader-to-reader interferences. LBTM protocol can be used together with these modes, adding some soft QoS control to them. In this section, we make a comparison of a LBTM+DIM operating protocol versus a standalone DIM network with the transmit mask of −30 dBch (henceforth, this parameter is called Adjacent Channel Power Rejection -ACPR-, following nomenclature from [50]) as specified in the standard [1].

First, we simulated a DIM configuration, where we assumed that each channel is serving a number *N* of interrogators (from 1 to 20). The scenario layout corresponds to the hospital scenario, given in Figure 7. Channel access times correspond to the ones specified in Figure 1. That is, the reader occupies the channel until the batch of tags is read, and then waits for 100 ms before accessing the channel again. The *N* active readers are changed randomly every 20 s, while the total simulated time is 8000 s. The transmission power of the readers is 0.1 W (0.5 W EIRP), and interference among readers is considered additive, taking into account the antenna directivity and distances among readers. The access is done with DFSA assuming perfect tag count estimation.

**Figure 13:**
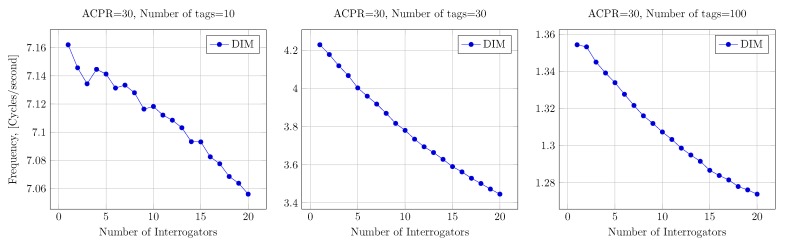
Degradation performance of Dense-Interrogation-Mode (DIM) with the number of nodes.

In this experiment, the average number of cycles that interrogators read batches per second is measured. Results are shown in Figure 13. While the aggregated interfering power of the readers is low due to the transmission mask applied, it can still cause a slight performance degradation. This effect is related to the increased number of misidentifications under the capture effect. That is, when the small additive noise coming from neighbor interrogators adds to the interfering noise in FSA slots accessed by several tags, the ratio of tags recovered by capture effect is reduced. This is the leading cause of the performance degradation observed in Figure 13.

**Figure 14:**
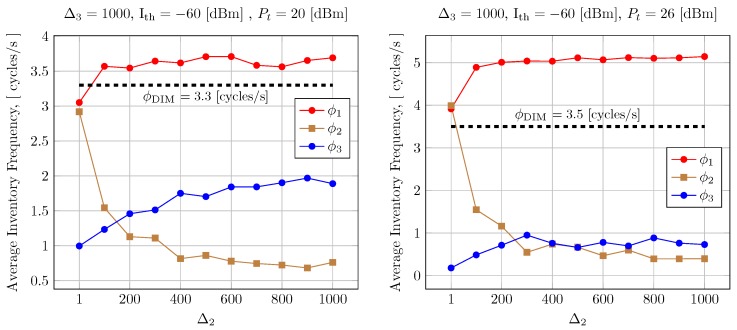
LBTM + DIM for 0.1-Watt and 0.4-Watt transmission power. Horizontal line is the DIM maximum reading frequency.

This result suggests that using some time multiplexing strategy between readers may result in an improved access rate for a selected set of readers because the global level of interferences will be kept controlled. Thus, we have applied LBTM for implementing this idea. When the reader senses power in the tags response channel over a given interference threshold ($I_{\mathrm{th}}$), then it delays its channel access (using the LBTM procedure). Results are shown in Figure 14 for the case of *N* = 20 readeres. The figure also contains the standalone DIM method performance ($\phi_{\mathrm{DIM}}$).

Summing up, LBTM can improve the operation of priority readers, also when used in combination with DIM. This is, of course, done at the expense of worsening the other traffics.

## 9. Conclusions

Results have shown that LBTM can trade-off the different performance indicators designed for the grocery store and hospital examples. LBTM has been designed as an easy to implement modification of the standard LBT, with additional back-off time before the first contention period, but with a normal operation afterward. This mechanism has been designed to avoid low-priority readers’ starvation. Results have shown that it is possible to adapt the network to different traffics by controlling the back-off delay properly with a unique parameter per reader (the Δ parameter). Similar benefits can be achieved in combination with DIM or MIM modes.

Moreover, we also developed an online reader configuration feature, allowing the reader to self-configure its Δ parameter by adapting it to achieve a local utility within a given target range. This allows readers to support dynamically changing traffic patterns, as well as network modifications.

## Figures and Tables

**Table 1:** 865.7 MHz tag portal example parameters in a linear scale. Full description is in [30].

$P_{T}$ (mW)	$G_{T}$, $G_{R}$	$G_{t}$	$X_{f},\;X_{b}$	λ (m)	*M*	τ	Θ	Sensitivity (dBm)
Transmitter Power	Transceiver Gains	Tag Gain	Polarization Mismatch	Wave-length	Modulation Factor	Power Coefficient	On-object Gain Penalty	Receiver Sensitivity
500	5	1.62	0.5	0.35	0.25	1	1.2	−80

**Table 2:** Utility parameters. $\Delta^{*}$ are the Δ’s of the optimal utility.

Traffic Class	a	b	$\phi_{\min}$ (cycles)	$\phi_{\max}$ (cycles)	$u(\phi^{*})$	$\Delta^{*}$	Notes
Patient monitoring	8.78	10.98	1	1.5	0.82	1	Reference frequencies are selected to achieved a number of cycles as high as possible and of at least 1 cycle/s
Doors	14.64	5.12	0.2	0.5	0.95	220	Reference frequencies aim at getting at least 1 interrogation opportunity for a maximum walking speed of 6 km/h considering a 2-m interrogation radius around door
Room inventorying	43.94	6.59	0.1	0.2	0.97	750	Reference frequencies aim at detecting critical equipment transfers in less than 1 min
